# Gestational Diabetes Mellitus and Associated Factors in Rural Areas of Northern Vietnam: Cross-Sectional Survey

**DOI:** 10.2196/79688

**Published:** 2026-03-31

**Authors:** Duc Cuong Le, Thi Hien Nguyen, Thi Ly Nguyen, Thi Nuong Tran, Xuan Hien Luong, Thi Thu Huong Do, Xuan Thuy Tran, Van Manh Ngo, Thi Huyen Dieu Bui, Tien Van Nguyen, Van Thuan Hoang, Thanh Binh Vu

**Affiliations:** 1 Thai Binh University of Medicine and Pharmacy, 373 Ly Bon Street, Thai Binh, Vietnam, 84 02273838545

**Keywords:** gestational diabetes mellitus, obesity, diabetes, risk factors, pregnant women

## Abstract

**Background:**

The prevalence of gestational diabetes mellitus (GDM) and its associated factors have not been investigated in Vietnam, especially in rural areas.

**Objective:**

This study aimed to determine the prevalence of GDM and its associated factors in rural areas of Vietnam.

**Methods:**

This cross-sectional study was conducted among 1003 pregnant women aged ≥18 years in rural areas of northern Vietnam. GDM was identified based on impaired oral glucose tolerance test results according to the guidelines of the International Association of the Diabetes and Pregnancy Study Groups. Associated factors for GDM were analyzed using a modified Poisson regression with robust (sandwich) SE analysis, with maternal age, prepregnancy BMI, and family history of diabetes as explanatory factors, adjusted for parity, method of conception, hormonal therapy for pregnancy maintenance, physical inactivity, and history of chronic medical conditions.

**Results:**

The prevalence of GDM was 26.2%. Patients with GDM were more likely to be older than the control group (odds ratio 3.33, 95% CI 2.31‐4.78). In the multivariable analysis, maternal age was strongly associated with GDM. Compared with women aged <25 years, those aged 25 to 34 years had a significantly higher prevalence of GDM (adjusted prevalence ratio [PR] 1.50, 95% CI 1.12‐1.99; *P*=.006). The rate was even higher among women aged ≥35 years (adjusted PR 2.40, 95% CI 1.74‐3.31; *P*<.001). These associations remained consistent after further adjustment for confounders (25‐34 years: adjusted PR 1.44, 95% CI 1.07‐1.95; *P*=.02 and ≥35 years: adjusted PR 2.15, 95% CI 1.49‐3.11; *P*<.001). Overweight women (BMI 23 to <25 kg/m²) showed a borderline association with GDM, although this did not reach statistical significance (adjusted PR 1.39, 95% CI 0.98‐1.98; *P*=.06), while women with BMI ≥25 kg/m² had a significantly higher prevalence (adjusted PR 1.58, 95% CI 1.10‐2.26; *P*=.01). These findings persisted in the adjusted model, with BMI ≥25 kg/m² remaining significantly associated with GDM (adjusted PR 1.54, 95% CI 1.10‐2.17; *P*=.01). Women with a family history of diabetes had an increased prevalence of GDM in both models, although the association did not reach statistical significance (adjusted PR 1.51, 95% CI 0.85‐2.68; *P*=.16). Most additional covariates included in the multivariable analysis were not significant. However, women who conceived via in vitro fertilization had a significantly higher prevalence of GDM compared with those who conceived naturally (adjusted PR 1.38, 95% CI 1.04‐1.85; *P*=.03). The use of hormonal therapy to maintain pregnancy was also associated with an increased risk (adjusted PR 1.32, 95% CI 1.04‐1.68; *P*=.02).

**Conclusions:**

The findings highlight the need for early screening and counseling before pregnancy. Lifestyle programs should focus on weight control and healthy habits. Future research should test whether these programs can reduce the rate of GDM in rural communities.

## Introduction

Gestational diabetes mellitus (GDM) is the most common metabolic disorder in pregnancy, affecting approximately 16.5% of pregnancies worldwide, and has become an increasingly major global public health concern because of its adverse implications for maternal and fetal health [1-3]. GDM is defined as any degree of glucose intolerance with the onset of or first recognition during pregnancy [4]. According to the report of the International Diabetes Federation, almost 21.3 million live births to women, or 16% of live births, were affected by some forms of high blood glucose in pregnancy. Moreover, the vast majority of pregnant women with GDM are in low- and middle-income countries, where access to maternal care is often limited [5,6]. Pregnant women exposed to maternal hyperglycemia from 24 to 28 weeks of gestation onward, if left undiagnosed and untreated, may experience serious short- and long-term outcomes for both mothers and their infants. GDM may cause pregnancy complications, such as preeclampsia or eclampsia or low blood sugar, and tends to be a major risk factor for the development of type 2 diabetes that develops later in life [7-9]. Therefore, identifying the prevalence of GDM and understanding the associated factors for GDM will offer the opportunity to improve pregnancy outcomes and maternal health as well as to drive health-promoting messages about diet and lifestyle.

The prevalence of GDM varies widely across countries, largely due to the use of different diagnostic criteria for GDM. The most recent cross-sectional study and meta-analysis reported that the global prevalence of GDM was 35% based on the new World Health Organization (WHO) diagnostic criteria for GDM and 14.7% based on the International Association of Diabetes and Pregnancy Study Groups (IADPSG) criteria [10,11]. In Lima, Peru, approximately 16% of pregnant women were diagnosed with GDM based on the IADPSG criteria [12]. In 2019, a meta-analysis using the same criteria reported that the prevalence of GDM in China was 14.8% [13]. Another recent systematic review and meta-analysis reported that the pooled prevalence of GDM ranged from 9% to 16%, with higher prevalence observed among pregnant women in urban populations [14].

The development of GDM in pregnant women has been associated with several factors. Previous studies have consistently identified advanced maternal age, obesity, and a family history of type 2 diabetes as major risk factors for GDM compared with women without these characteristics [3,15,16]. Other associated factors for GDM include a history of unexplained stillbirth [16].

Vietnam is undergoing a rapid epidemiologic transition characterized by rising rates of overweight, obesity, and noncommunicable diseases, including diabetes. Several studies from urban or periurban regions of Vietnam have reported GDM prevalence estimates ranging from 7% to 25% depending on diagnostic criteria [11,17]. However, data on GDM burden in rural areas, where access to maternal health services is more limited, remain limited [18,19]. Rural populations face unique challenges, including limited screening capacity, lower health literacy, and substantial socioeconomic disparities, which may contribute to underdiagnosis or delayed detection of GDM. Updated evidence on the magnitude and associated factors of GDM in rural Vietnam is needed to inform targeted interventions and maternal health policies. Therefore, we conducted this study to determine the prevalence of GDM and identify key maternal factors associated with GDM among pregnant women living in rural areas of northern Vietnam.

## Methods

### Participants

This cross-sectional study was conducted in Thai Binh province from June 2021 to May 2022. It is located approximately 100 km north of Hanoi, the capital city of Vietnam. The economy is mostly agricultural. The total population is 1.86 million people, the gross domestic product per capita is US $1650, and more than 90% of the population lives in rural areas. Thai Binh province is subdivided into 7 districts and 1 provincial city. The study was conducted at 3 hospitals in a provincial city, including Medicine University, Provincial General, and Gyneco-Obstetrics Hospital.

### Recruitment Procedures

Recruitment was conducted at the antenatal outpatient clinics of the 3 study hospitals. These clinics provide routine antenatal services, including pregnancy monitoring, screening for obstetric complications, counseling, and laboratory testing, including oral glucose tolerance testing for GDM.

A systematic and consecutive recruitment approach was applied. During the study period, all pregnant women attending antenatal clinics were screened for eligibility. Women were eligible if they were aged 18 years or older and at 24 to 28 weeks of gestation, which corresponds to the routine screening period for GDM in Vietnam. Women with a known diagnosis of diabetes mellitus prior to pregnancy were excluded.

Eligible women were consecutively approached by trained research staff during their routine antenatal visit. Recruitment was conducted on all clinic working days to minimize selection bias. The study objectives, procedures, potential risks, and benefits were explained verbally in Vietnamese, the native language of the participants. Women were given sufficient time to ask questions and consider participation.

### Questionnaire

The pregnant women were interviewed using a questionnaire constructed in Vietnamese, the native language of the participants. Information on the following potential associated factors was collected during this survey: women’s age in years; women’s education level (high school or below or upper high school); women’s occupation (housewife, farmer, or small business; worker; state officer; private officer; or others); family history of diabetes (yes or no); parity (nulliparous or multiparous); pregnancy method (natural conception or in vitro fertilization [IVF]); height and weight in the prepregnancy period to calculate BMI; hormonal therapy for pregnancy maintenance; limited movement and exercise, defined as less than 3 hours of physical exercise per week; and medical history of any chronic disease.

### GDM Diagnostic Criteria

GDM was diagnosed according to the guidelines of the IADPSG using the 75-g oral glucose tolerance test with 3 venous blood samples. The criteria included diagnosis based on any of the following three values of plasma glucose: (1) fasting blood glucose level of 5.1 to 6.9 mmol/L (92‐125 mg/dL), (2) 1-hour blood glucose level of ≥10.0 mmol/L (180 mg/dL), and (3) 2-hour blood glucose level of 8.5 to 11.0 mmol/L (153‐199 mg/dL).

### Statistical Analysis

The data were entered using Epidata (version 3.1; EpiData Association) software. Stata (version 15.0; StataCorp LLC) software was used for statistical analysis. General characteristics of the study participants were presented as numbers and percentages. Chi-square or Fisher exact tests were conducted to compare the differences in proportion between 2 groups of patients.

The primary outcome was GDM, defined as a binary variable (yes or no). The main explanatory variables included maternal age (<25, 25‐34, and ≥35 years), family history of diabetes (yes or no), and prepregnancy BMI, which was categorized according to the classification for Asian populations (<23, 23-<25, and ≥25 kg/m²) [20]. The selection of these variables was based on previous studies [11,12,15,21].

Associations between each explanatory variable and GDM were first explored using univariable analysis to estimate crude prevalence ratios (crude PRs) and 95% CI. Subsequently, a multivariable modified Poisson regression model with robust (sandwich) SEs (model 1) was constructed to assess the association between explanatory factors and GDM. A second model (model 2) was fitted to further adjust for potential confounders identified a priori based on clinical relevance and previous literature. These included parity (nulliparous vs multiparous), method of conception (natural vs IVF), hormonal therapy for pregnancy maintenance (yes or no), physical inactivity (defined as <3 hours of exercise per week), and history of chronic medical conditions (yes or no). The results from both model 1 and model 2 were presented as adjusted prevalence ratios (adjusted PRs) with corresponding 95% CIs. A *P* value of <.05 and a 95% CI were chosen as the statistical significance level and CI, respectively.

### Ethical Considerations

The study was conducted in accordance with the Declaration of Helsinki and approved by the Institutional Review Board of the Thai Binh Department of Science and Technology (protocol code 1369/QĐ-UBND, approved on June 10, 2021) for studies involving humans. All participants provided oral informed consent. Participation in the study was completely voluntary. Participants were free to withdraw from the study at any time without any consequences for their medical care. The data were protected and deidentified before analysis. No financial or material compensation was provided to participants.

## Results

### Overview

**Figure 1.**
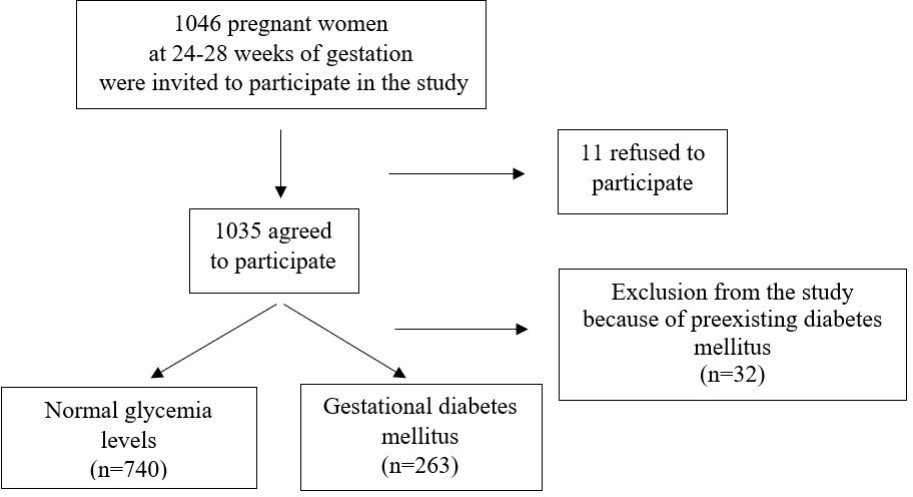
Study flow diagram.

A total of 1046 pregnant women at 24 to 28 weeks of gestation were invited, and 11 (1.1%) refused to participate. In addition, 32 (3.0%) were excluded because of preexisting diabetes mellitus. Of the 1003 (95.9%) included women, 263 were diagnosed with GDM; hence, the GDM proportion was 26.2% (Figure 1).

### Characteristics of Participants

Table 1 presents the characteristics of the participants stratified according to GDM status, including sociodemographic characteristics and clinical characteristics of participants. A proportion of 22.8% (60/263) of women with GDM were aged ≥35 years, compared with 10% (74/740) of women without GDM (*P*<.001). In addition, the prevalence of GDM was significantly higher among women with a BMI ≥25 kg/m^2^, a family history of diabetes, a history of 2 or more pregnancies, conception via IVF, and use of endocrine medicines, compared with women without GDM. There was no difference in blood pressure classification between the 2 groups (Table 1).

**Table 1.** Background characteristics of participants.

Characteristics	Gestational diabetes mellitus, n (%)		*P* value^a^
	Yes (n=263)	No (n=740)	
Age (years)			<.001
<25	50 (19.0)	240 (32.4)	
25‐34	153 (58.2)	426 (57.6)	
≥35	60 (22.8)	74 (10.0)	
Educational background (levels)			.16
High school and below	97 (36.9)	310 (41.9)	
Upper high school	166 (63.1)	430 (58.1)	
Occupations			.74
Housewife, farmer, or small business	70 (26.6)	203 (27.4)	
Worker	58 (22.1)	171 (23.1)	
State officer	31 (11.8)	105 (14.2)	
Private officer	90 (34.2)	227 (30.7)	
Others	14 (5.3)	34 (4.6)	
BMI (kg/m^2^)			.002
<23	223 (84.8)	682 (92.2)	
23 to <25	23 (8.7)	35 (4.7)	
≥25	17 (6.5)	23 (3.1)	
Family history of diabetes			.02
No	255 (97.0)	731 (99.1)	
Yes	8 (3.0)	7 (0.9)	
Parity			.003
Nulliparous	122 (46.4)	422 (57.0)	
Multiparous	141 (53.6)	318 (43.0)	
Pregnancy method			<.001
Natural	227 (86.3)	684 (94.5)	
In vitro fertilization	36 (13.7)	40 (5.5)	
Hormonal therapy for pregnancy maintenance			<.001
Yes	86 (35.5)	143 (20.7)	
No	156 (64.5)	547 (79.3)	
Limited movement and exercise (<3 hours of physical exercise per week)			.31
Yes	63 (24.0)	155 (21.0)	
No	200 (76.0)	585 (79.0)	
Medical history of chronic diseases			.47
Yes	17 (6.5)	39 (5.3)	
No	246 (93.5)	701 (94.7)	
Blood pressure (systolic and diastolic; mmHg)			.50
<120 and <80	469 (63.4)	1256 (59.3)	
120‐129 and <80	98 (13.2)	33 (12.6)	
130‐139 or 80‐89	151 (20.4)	65 (24.7)	
≥140 or ≥90	22 (3.0)	9 (3.4)	

a Fisher exact or chi-square test.

### Associated Factors for GDM

In the univariable analysis, maternal age, BMI, family history of diabetes, parity, pregnancy method, and hormonal therapy were significantly associated with GDM. Women aged 25 to 34 years and ≥35 years had higher prevalence of GDM compared with those aged <25 years (crude PR 1.53 and 2.60, respectively). Higher prepregnancy BMI increased the prevalence of GDM, with crude PRs of 1.60 for BMI 23 to <25 kg/m² and 1.72 for BMI ≥25 kg/m². A family history of diabetes was associated with a higher prevalence of GDM (crude PR 2.07). Multiparous women also had an increased prevalence of GDM (crude PR 1.37). IVF and use of hormonal therapy were strongly associated with GDM (crude PR 1.93 and 1.64, respectively). Limited physical activity and a history of chronic disease were not significantly associated with GDM (Table 2).

**Table 2.** Associated factors of gestational diabetes mellitus, univariate analysis.

Factors	Crude PR^a^ (95% CI)		*P* value
Age (years)			
<25	Reference		
25‐34	1.53 (1.15‐2.04)		.003
≥35	2.60 (1.90‐3.56)		<.001
BMI (kg/m^2^)			
<23	Reference		
23 to <25	1.60 (1.15‐2.26)		.006
≥25	1.72 (1.18‐2.52)		.005
Family history of diabetes			
No	Reference		
Yes	2.07 (1.27‐3.36)		.003
Parity			
Nulliparous	Reference		
Multiparous	1.37 (1.11‐1.69)		.003
Pregnancy method			
Natural	Reference		
In vitro fertilization	1.93 (1.49‐2.52)		<.001
Hormonal therapy for pregnancy maintenance			
No	Reference		
Yes	1.64 (1.33‐2.03)		<.001
Limited movement and exercise (<3 hours of physical exercise per week)			
No	Reference		
Yes	1.13 (0.89‐1.44)		.30
Medical history of chronic diseases			
No	Reference		
Yes	1.17 (0.77‐1.76)		.46

a PR: prevalence ratio.

In the multivariable analysis (model 1), maternal age was strongly associated with GDM. Compared with women aged <25 years, those aged 25 to 34 years had a significantly higher prevalence of GDM (adjusted PR 1.50, 95% CI 1.12‐1.99; *P*=.006). The prevalence was even higher among women aged ≥35 years (adjusted PR 2.40, 95% CI 1.74‐3.31; *P*<.001). These associations remained consistent in model 2 after further adjustment for confounders (25‐34 years: adjusted PR 1.44, 95% CI 1.07‐1.95; *P*=.02 and ≥35 years: adjusted PR 2.15, 95% CI 1.49‐3.11; *P*<.001).

Overweight women (BMI 23 to <25 kg/m²) showed a borderline association with GDM, although this did not reach statistical significance (adjusted PR 1.39, 95% CI 0.98‐1.98; *P*=.06), while women with BMI ≥25 kg/m² had a significantly higher prevalence (adjusted PR 1.58, 95% CI 1.10‐2.26; *P*=.01). These findings persisted in model 2, with BMI ≥25 kg/m² remaining significantly associated with GDM (adjusted PR 1.54, 95% CI 1.10‐2.17; *P*=.01).

Women with a family history of diabetes had an increased prevalence of GDM in both models, although this did not reach statistical significance (model 1: adjusted PR 1.78; 95% CI 1.01‐3.14; *P*=.04 and model 2: adjusted PR 1.51, 95% CI 0.85‐2.68; *P*=.16).

Most additional covariates included in model 2 were not significant. However, women who conceived via IVF had a significantly higher prevalence of GDM compared with those who conceived naturally (adjusted PR 1.38, 95% CI 1.04‐1.85; *P*=.03). The use of hormonal therapy to maintain pregnancy was also associated with an increased prevalence (adjusted PR 1.32, 95% CI 1.04‐1.68; *P*=.02; Table 3).

**Table 3.** Modified Poisson regression model with robust (sandwich) SEs analysis of factors associated with gestational diabetes mellitus.

Associated factors	Model 1^a^		Model 2^b^	
	Adjusted PR^c^ (95% CI)	*P* value	Adjusted PR (95% CI)	*P* value
Age (years)				
<25	Reference		Reference	
25‐34	1.50 (1.12‐1.99)	.006	1.44 (1.07‐1.95)	.02
≥35	2.40 (1.74‐3.31)	<.001	2.15 (1.49‐3.11)	<.001
BMI (kg/m^2^)				
<23	Reference		Reference	
23 to <25	1.39 (0.98‐1.98)	.06	1.34 (0.94‐1.92)	>.99
≥25	1.58 (1.10‐2.26)	.01	1.54 (1.10‐2.17)	.01
Family history of diabetes				
No	Reference		Reference	
Yes	1.78 (1.01‐3.14)	.04	1.51 (0.85‐2.68)	.16
Parity				
Nulliparous	—^d^	—	Reference	
Multiparous	—	—	0.99 (0.78‐1.25)	.93
Pregnancy method				
Natural	—	—	Reference	
In vitro fertilization	—	—	1.38 (1.04‐1.85)	.03
Hormonal therapy for pregnancy maintenance				
No	—	—	Reference	
Yes	—	—	1.32 (1.04‐1.68)	.02
Limited movement and exercise (<3 hours of physical exercise per week)				
No	—	—	Reference	
Yes	—	—	0.94 (0.75‐1.91)	.62
Medical history of chronic diseases				
No	—	—	Reference	
Yes	—	—	0.96 (0.65‐1.42)	.83

a Model 1 includes maternal age, prepregnancy BMI, and family history of diabetes.

b Model 2 includes all variables in model 1 plus parity, pregnancy method, hormonal therapy for pregnancy maintenance, limited physical activity (<3 hours per week), and medical history of chronic diseases.

c PR: prevalence ratio.

d Not applicable.

## Discussion

### Principal Findings

This cross-sectional survey provides estimates of the prevalence of GDM among pregnant women, as well as the associations between specific demographic characteristics and GDM, in rural areas of Vietnam. To the best of our knowledge, this is the first study to examine the prevalence of GDM by the IADPSG criteria in Vietnam. In this cross-sectional study of 1003 pregnant women in rural northern Vietnam, we found that the prevalence of GDM was 26.2%. Consistent with the study objectives, several sociodemographic and clinical factors were identified as significantly associated factors for GDM. Older maternal age demonstrated a clear dose-response relationship, with women aged ≥35 years having the highest odds of GDM. Prepregnancy overweight and obesity (BMI ≥23 kg/m²) were also strongly associated with increased GDM risk. A family history of diabetes showed a positive but borderline association.

This prevalence was higher than that reported in previous studies. For instance, the overall prevalence of GDM among study participants in Ghana was 8.5% [22], and in Lima, it was approximately 16% [12]. In Eastern and Southeast Asia, the prevalence of GDM was 11.91% in China, while in Japan, Korea, and Thailand, it was less than 8.0% [23]. A recent prospective cohort study showed that the incidence and age-adjusted incidence of GDM in China were 17.42% and 17.45%, respectively [24].

In this study, we observed that maternal age is an associated factor for GDM. This finding is consistent with studies by Lin et al [25] and Li et al [24], which reported that older maternal age was associated with a higher risk of developing GDM compared with women younger than 35 years. The underlying mechanism for this association may be attributed to hormonal changes that occur as women age, leading to alterations in glucose and insulin processing and consequently raising the risk of GDM during pregnancy. Therefore, this study reinforced the findings of previous studies and indicated that pregnancy is better planned before the age of 35 years, and it is crucial to implement a regular screening plan for GDM to detect and manage the condition effectively, thus ensuring optimal health outcomes for both the mother and the baby.

Our study also showed that pregnant women with BMI ≥25 kg/m² had a significantly higher prevalence of GDM compared with women of normal weight. In addition, those with a BMI of 23 to 25 kg/m² demonstrated a trend toward an increased prevalence of GDM, suggesting a graded association across Asian-specific BMI categories. Although the association in this intermediate BMI category did not reach conventional statistical significance, it represents a clinically important early warning stage, rather than a neutral risk group. This finding is consistent with previous studies [3,12,15,16] and supports the use of lower BMI cutoffs for early risk identification. From a clinical and public health perspective, this finding is particularly important in rural settings, where women with BMI of 23 to 25 kg/m² are often not identified as high risk and may not receive targeted counseling or early screening. Our results suggest that this BMI range could serve as a practical early warning threshold, offering an opportunity for lifestyle modification, dietary counseling, and closer metabolic monitoring to prevent progression to overt obesity and clinically manifest GDM. Overweight and obesity are closely linked to excessive and prolonged calorie intake, which might overwhelm pancreatic β-cell insulin production and insulin signaling pathways. Even independent of BMI, diet and nutrition also play an important role in the development of GDM. Diets high in saturated fat, refined sugar, and red or processed meat have been associated with an increased risk of GDM [3], while diets high in fiber, micronutrients, and polyunsaturated fats are consistently associated with a reduced risk of GDM. Saturated fats directly interfere with insulin signaling. In addition, they can also cause inflammation and endothelial dysfunction. These are the 2 main factors that cause GDM [3].

Women who conceived after IVF had a higher prevalence of GDM in our study. This result suggests that IVF pregnancy is an independent associated factor for GDM in this rural population. The finding is consistent with many previous studies [26-28]. These analyses reported that the proportion of GDM after assisted reproductive technology was often approximately twofold higher. A systematic review and meta-analysis also showed that assisted reproductive technology is associated with about a 1.5-fold increase in GDM [29]. The magnitude of effect in our study is similar to that in the literature. This supports the idea that IVF itself, or factors linked to IVF, may contribute to disturbed glucose metabolism in pregnancy. Several mechanisms may explain this association. Women who need IVF often have older age or long-standing infertility. Many have conditions such as polycystic ovary syndrome or obesity. These factors increase insulin resistance and baseline diabetes risk. Supraphysiologic hormone levels during ovarian stimulation may further worsen insulin resistance [30]. IVF pregnancies also receive closer surveillance. This can increase the detection of milder GDM. Our models were already adjusted for age, BMI, and parity. However, residual confounding by unmeasured fertility-related factors may still be present.

The use of hormonal therapy to maintain pregnancy was also associated with GDM in our study. Indeed, progesterone and other progestogens reduce insulin sensitivity and are part of the hormonal milieu that drives insulin resistance in pregnancy [31]. Meta-analyses on progestogen supplementation to prevent preterm birth suggest a possible increase in GDM risk [32]. In addition, hormonal therapy for pregnancy maintenance likely marks a subgroup of women with threatened miscarriage, luteal phase problems, or other endocrine disorders. These women may already carry a higher metabolic risk.

These findings have important clinical implications. IVF conception and the use of hormonal therapy can be viewed as simple clinical markers for higher GDM risk. Women who receive these treatments may benefit from early glucose screening, closer metabolic follow-up, and strong counseling on weight control and physical activity. Preconception assessment in fertility clinics could also help to identify impaired glucose tolerance before pregnancy.

Although the association was not significant, our study also suggested that a family history of diabetes may be a potential risk factor for GDM. Similarly, Lin et al [25] and Rhee et al [33] reported that a family history of diabetes is a major risk factor for GDM in Asian populations. This may be explained by the fact that women with GDM often carry susceptibility genes for type 2 diabetes [34]. Therefore, pregnant women with a family history of diabetes should focus on weight management, regular exercise, healthy diet, and monitoring blood sugar levels to prevent GDM. However, the association between obstetric history and family history of diabetes with GDM was only observed in our univariate analysis.

Some limitations must be considered when interpreting the results of our study. First, the use of a cross-sectional study design limits the findings of this study, as causal relationships cannot be established. Second, family history of diabetes was assessed based on self-report; therefore, we cannot rule out recall bias. Additionally, participants in this study were pregnant women living in Thai Binh, which may limit the generalizability of our findings to other populations. Moreover, our study relied on self-reported lifestyle changes and did not assess adherence to these changes, which may have influenced the outcomes. Finally, the study did not include important socioeconomic indicators. We did not collect data on income, living conditions, access to health services, or socioeconomic status. These factors may influence the risk of GDM, especially in rural settings. Their absence may limit the interpretation of our results.

### Conclusions

Our study found that maternal age ≥35 years and prepregnancy obesity were associated with an increased prevalence of GDM. Women who conceived through IVF or used hormonal therapy also had higher rates of GDM. These results show that rural women face important metabolic risks during pregnancy. The findings highlight the need for early screening and counseling before pregnancy. Lifestyle programs should focus on weight control and healthy habits. Future research should test whether these programs can reduce the rate of GDM in rural communities.

## Abbreviations

GDM: gestational diabetes mellitus
IADPSG: International Association of Diabetes and Pregnancy Study Groups
IVF: in vitro fertilization
PR: prevalence ratio
WHO: World Health Organization
